# Reply: 18S is an appropriate housekeeping gene for *in vitro* hypoxia experiments

**DOI:** 10.1038/sj.bjc.6605755

**Published:** 2010-07-20

**Authors:** J Caradec, N Sirab, C Keumeugni, D Revaud, S Loric

**Affiliations:** 1 INSERM, U955 EQ07, Créteil, France; 2 Paris Est University, Créteil, France; 3 APHP Clinical Biochemistry and Genetics Department, Mondor University Hospital, Créteil, France

**Sir,**

We have read with great attention the letter sent by Nagelkerke *et al.* This team has in the past published one of the most interesting articles on housekeeping gene (HKG) variations (de Kok *et al*, 2005). On the basis of their own previous laboratory experience and specific data obtained on hypoxia experiments (Mujcic *et al*, 2009), they reported that whether 18S rRNA was not stable enough to be used to normalise results in human tissues, it seemed to be a good HKG in cell lines to compensate input, reverse transcription, and PCR efficiency. As 18S rRNA was not one of our selected HKGs in our study, they rightly suggested studying its expression on our hypoxic cDNA bank. For this purpose, we used the same 18S primers they used in their trials (18S_W: F-agtccctgccctttgtacaca and R-gatccgagggcctcactaaac) and performed quantitative real-time PCR (qRT-PCR), as described earlier (Caradec *et al*, 2010), on four prostate (PNT2 and LNCaP), kidney (HEK) and breast (MCF-7) cell lines treated with four oxygen concentrations (1, 5, 10, and 20%).

Results first show that 18S amplification is constantly detected at around 15 PCR cycles threshold (*C*_t_). As to cell variability, r18S expression seems to be relatively stable (coefficient of variation (*C*_t_CV%) <3%) in three cell lines, except in LNCaP prostate tumor cell line where *C*_t_CV% is above 6%. These results have been confirmed using a newly designed set of 18S specific primers (18S_C: F-catggccgttcttagttggt and R-cgctgagccagtcagtgtag) (Figure 1). When 18S results were compared with those we already obtained with other HKG (Caradec *et al*, 2010), we found that 18S appears to be the best one in HEK cell line and the second best one in PNT2 cell lines (Table 1). However, it is likely to be one of the worst in LNCaP and MCF-7.

Our study results do neither corroborate nor contradict the conclusions raised by Nagelkerke *et al.* They reported r18S as the best HKG to normalise results in hypoxia experiments, and we found that it may be a good one in two out of the four cell lines we studied in hypoxia conditions. Whereas r18S could be a good and reasonable choice for some cell lines, it would not be the right one for others. As stated by Nagelkerke *et al*, this holds also true for *in vivo* systems as the compensation system that is convenient for a dedicated *in vitro* model is not the one necessarily recommended when tissues have to be studied. That is why we persist in arguing that every HKG must be tested before experiments to choose the best one: this necessary step will determine result's reliability, and should be performed for every new study design. As we tested r18S, an important issue has to be discussed here regarding its use as a dedicated compensate for input, RT and PCR efficiency. Indeed, most of target transcripts of interest are expressed at a largely lower level than r18s (generally over 25 up to 35–36 *C*_t_ – over this limit, results are likely to be uninterpretable). As a difference of 10–15 *C*_t_ corresponds to a difference of 10^3^- to 10^5^-folds in expression level, it remains to establish whether a highly expressed gene could serve to normalise weakly expressed transcripts. There are no clear evidences that transcripts present at large number of copies are extracted at the same yield level as scarser ones. Moreover, it could also be the same for cDNA production as it is tempting to speculate that reverse transcriptase may be more efficient when large amount of substrate is present than for tiny represented targets that have a stochastic probability to be similarly processed.

As RNA extraction protocols, as well as RT or PCR efficiencies remained strongly laboratory reagents, protocols, and instrument dependents, worldwide standardisation of these techniques is still strongly mandatory. As these standards are not available yet, using HKG remains an interesting option that presupposes that HKGs have been carefully tested and controlled in the experimental system they have to be used.

## Figures and Tables

**Figure 1:**
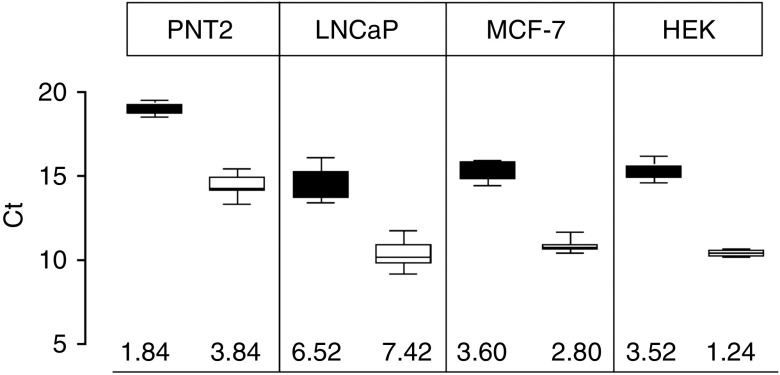
r18S variations in PNT2, MCF-7, LNCaP, and HEK cell lines grown in different hypoxic conditions (1, 5, 10, and 20% oxygen). Box plots represent cycle threshold (*C*_t_) variations measured with different primers (black box: 18S_W, white box: 18S_C, value representing *C*_t_CV% is indicated below corresponding box plot). r18S expression was quantified by qRT-PCR in duplicates in three independent experiments.

**Table 1:** Housekeeping genes ranking according to their stability in each cell line

	***PNT2***	***LNCaP***	***MCF-7***	***HEK***
18S_C	6	12	10	1
18S_W	2	11	12	2
ACTB	9	4	5	12
ATP5G3	1	5	3	10
B2M	12	3	9	7
GAPDH	8	2	11	4
GUSB	11	8	4	3
HRPT1	4	7	8	8
PGK1	7	9	9	5
PPIA	10	10	7	9
TBP	5	1	1	11
TFRC	3	6	2	6
